# Hypoxia-induced MFAP5 Promotes Tumor Migration and Invasion via AKT Pathway in Head and Neck Squamous Cell Carcinoma

**DOI:** 10.7150/jca.38217

**Published:** 2020-01-14

**Authors:** Qiaoshi Xu, Hanyue Chang, Xuerui Tian, Chao Lou, Hailong Ma, Xi Yang

**Affiliations:** 1 Department of Oral Maxillofacial-Head and Neck Oncology, Shanghai Ninth People's Hospital, College of Stomatology, Shanghai Jiao Tong University School of Medicine, No 639, Zhizaoju Rd, Shanghai 200011, China; 2 National Clinical Research Center for Oral Diseases, Shanghai 200011, China.; 3 Shanghai Key Laboratory of Stomatology & Shanghai Research Institute of Stomatology, Shanghai 200011, China.; 4 Key Laboratory of Oral Medicine, Guangzhou Institute of Oral Disease, Stomatology Hospital of Guangzhou Medical University, Guangzhou, 510140, China.

**Keywords:** Microfibrillar-associated protein 5, Head and neck squamous cell carcinoma, Migration, Invasion, AKT

## Abstract

**Objective**: Microfibrillar-associated protein 5 (MFAP5) is highly expressed in many types of cancers. Our previous study has observed that overexpression of MFAP5 was correlated with lymph nodes metastasis and poor prognosis in head and neck squamous cell carcinoma (HNSCC), but the underlying mechanism is poorly understood.

**Materials and methods**: The MFAP5 expression is detected under hypoxia condition. HNSCC cell lines are transfected with MFAP5-expressing lentivirus vector to establish stable overexpression model. Wound-healing, migration and invasion assay are used to determine the effect of MFAP5 on HNSCC and metastasis-related proteins are examined by Western blot. *In vivo* lung metastasis assays are conducted by the tail vein injection. In addition, immunohistochemistry is applied to analyze the correlation of MFAP5, hypoxia-induced factor-1 α (HIF-1α), and vimentin in 84 HNSCC patients' tissue samples.

**Results**: Firstly, MFAP5 expression can be markedly induced under hypoxia condition in HNSCC cell lines. Cell lines with MFAP5 overexpression has a significant higher ability of migration and invasion. In addition, *in vivo* assay observes that overexpression of MFAP5 can promote tumor lung metastasis. Furthermore, MFAP5 facilitates this process by activating epithelial-mesenchymal transition (EMT) program via AKT pathway in HNSCC cell lines. The pro-metastatic effect of MFAP5 can be reversed by MK2206, an AKT phosphorylation inhibitor. Lastly, the positive correlation among HIF-1α, MFAP5 and vimentin from tissue samples and TCGA dataset are also observed in HNSCC.

**Conclusion**: Our study demonstrates MFAP5 plays a critical role in hypoxia-induced EMT program via AKT pathway in HNSCC, which would be a very promising therapeutic target.

## Introduction

Head and neck squamous cell carcinoma (HNSCC) is one of the most common cancers and affects more than 600,000 patients per year 1. Despite large advances in cancer treatment strategies, the long-term survival of HNSCC patients remain poor. According to recent studies, tumor recurrence and metastasis are the major causes of death among cancer patients 2-4. The study on mechanisms of invasion and metastasis of tumor cells receive more and more considerations.

Microfibrillar-associated protein 5 (MFAP5), as known as Microfibril-associated glycoprotein 2 (MAGP2), has an important role in building microfibril networks and forming vascular structure 5-7. It has been proven as an oncogene in many types of cancers 8-10. Mok et al. claimed that MFAP5 can prolong tumor cell survival and stimulate endothelial cell motility in ovarian cancer 11; Li et al. demonstrated that knockdown MFAP5 can markedly reduce the cell proliferation, migration and invasion depend on ROS production in cervical cancer 12. Other studies showed MFAP5 has obvious impact on epithelial-mesenchymal transition (EMT) progression 13, 14. In our previous study, we have observed that MFAP5 was overexpression in HNSCC and correlated with lymph nodes metastasis and poor prognosis 15. However, the biologic function and underlying mechanism of MFAP5 in HNSCC is lack of study.

Hypoxia is a common situation in many solid tumors and has been considered as a key part in tumor progression 16. Hypoxia-inducible factor-1α (HIF-1α), the direct responder of hypoxia, is related with poor prognosis and highly expressed in metastasis tumor 17. Accumulative evidences showed that hypoxia can initiate EMT progression via HIF-1α 18, 19. Interestingly, although both being a cancer-promoting factor, the relationship between hypoxia and MFAP5 is rarely explored. Moreover, MFAP5 is believed to be secreted mainly by tumor stroma cells 20. Whether MFAP5 can play a crucial role in hypoxia-induced EMT progression is still obscure.

In the present study, we found that hypoxia condition could induce MFAP5 expression by HNSCC cells which indicated that tumor cells are also an important resource of MFAP5. A series experiments demonstrated that MFAP5 promote tumor migration and invasion of HNSCC both* in vitro* and *in vivo* through AKT pathway. Overexpression of MFAP5 significantly correlated with advanced clinical stage, metastasis and poor prognosis of HNSCC patients. Our findings not only suggested that hypoxia environment could stimulate tumor cell to secret MFAP5 but also proved that MFAP5 promotes EMT program in HNSCC. These findings show the critical role of MFAP5 in hypoxia induced tumor progression, which would be a very promising therapeutic target.

## Materials and Methods

### Patients and tissue samples

This study was conducted in full accordance with ethical principles, and approved by the Medical Ethics Committee of the Ninth People's Hospital, Shanghai Jiao Tong University, School of Medicine. Patients' inclusion criteria included the following: (1) patients with a pathological diagnosis of squamous cell carcinoma; (2) patients who were primarily treated with surgery; (3) patients with no previous treatment; (4) patients with complete clinicopathological data and available tissue specimens. The exclusion criteria included preoperative chemotherapy or radiotherapy, failure to undergo surgery and the inability to obtain pathological slices. From January 2007 to December 2008, a total of 84 HNSCC patients were met the inclusion criteria. The tissue samples and medical records of patients enrolled were collected by strict procedures.

### Cell culture

The cell lines used in this study were Cal27 and HN30. Cal27 were purchased from ATCC (Manassas, VA). The cell lines HN30 was established from pharyngeal squamous cell carcinoma and were kindly provided by the University of Maryland Dental School, USA. All these cell lines were cultured in Dulbecco's modified Eagle's medium (DMEM) (Gibco, Carlsbad, CA) supplemented with 10% fetal bovine serum, 1% glutamine, and 1% penicillin-streptomycin. Cells were cultured in a standard humidified atmosphere of 5% CO2 at 37 °C in general and in hypoxia cell incubator (ESCO, Singapore) at 2% O2 for hypoxia experiment.

### Immunohistochemistry

The tissue samples of enrolled patients were examined for the expression of MFAP5, HIF-1α and vimentin by immunohistochemical staining. The staining followed the standard protocol. Briefly, paraffin-embedded sections were heated by water bath at 100 °C with citrate buffer solution (pH 6.0) for 20 minutes to retrieve antigen, and were cooled at room temperature. The primary antibodies were monoclonal antibody against MFAP5 (ab203828, Abcam, USA), HIF-1α (ab113642, Abcam, USA) and vimentin (D21H3, Cell signaling technology, USA) and were incubated overnight at 4 °C, then visualized using 3,3'-diaminobenzidine (DAB) detection kit (Dako Cytomation, Denmark) containing goat secondary antibody molecules and DAB chromogen. Every step of the wash used phosphate buffered saline solution (PBS) for 5 minutes three times. The intensity of the MFAP5, HIF-1α and vimentin immunoreaction was scored as following: 0 = negative, absence of stained cells; 1 = weak; 2 = moderate; 3 = strong. The immunohistochemical staining score was calculated by multiplying the percentage of positive cells and the staining intensity as described in the literature.

### Lentivirus transfection

For gene overexpression, lentivectors containing MFAP5 sequence or control lentivectors (Genomeditech, Shanghai, China) were transfected into Cal27 and HN30 cells according to the manufacturer's instruction. The transfected cells were treated with puromycin (5 μg/mL) for 2 weeks to establish stable cell lines.

### Transwell assay

For transwell assays, 2~10×10^4^ transfected cells were seeded in the upper chambers of filter inserts containing serum-free medium. The chambers without matrigel were used to detect cell migration ability while the one with matrigel to analyze cell invasion. 500 μl medium containing 10% FBS were held in the lower chambers in 24-well plates. After incubation of 48 h, the cells migrated through the membrane were fixed, stained with 5% crystal violet. The number of cells and photographs were counted and taken with a Nikon DMCI microscope (Nikon, Tokyo, Japan).

### Wound healing assay

Briefly, transfected cells were seeded in a 6-well plate overnight and yielding confluent monolayers suitable for wounding. The wounds were scratched by a 10 μl pipette tip and photographs were taken at 0 h and 24 h. The width of wound was measured to assess the migration ability of cells.

### Real-time PCR

Total RNA was extracted from cultured cells with Trizol Reagent (Invitrogen) following the manufacturer's protocol. RNA was reversely transcribed to cDNA using the SYBR Premix Ex Taq reagent kit (Takara, Japan). Real-time PCR (qRT-PCR) was performed using a StepOnePlus Real-time PCR system (Thermo Fisher, Waltham, MA, USA) using the following specific primers: MFAP5: forward: 5′-TGGCTGATATTGCACCTTCCAC-3' and reverse: 5′-ATTTCTCATCCCAGCACTCTGC-3'; GAPDH forward: 5′-CCTCTGACTTCAACAGCGAC-3′ and reverse: 5′-TCCTCTTGTGCTCTTGCTGGC-3′.

### Western Blot

Western blot assay was performed using the standard method as briefly described 21,22. Protein samples were collected after cells were lysed and quantified. 20~30 μg/lane protein were separated by 10%~15% sodium dodecyl sulfate-polyacrylamide gel electrophoresis (SDS-PAGE). A wet transfer (Bio-Rad, USA) were then performed to transfer the protein onto polyvinylidene fluoride (PVDF) membranes and blocked with non-fat milk for 1 h. The membranes were incubated with primary antibody MFAP5 (HPA010553,Sigma,Germany), MMP9 (D6O3H, Cell signaling technology, USA), MMP2 (D4M2N Cell signaling technology, USA), vimentin (D21H3, Cell signaling technology, USA), Sanil (C15D3, Cell signaling technology, USA), AKT (11E7, Cell signaling technology, USA), p-AKT (D9E, Cell signaling technology, USA), HIF-1α (D1S7W, Cell signaling technology, USA), α-tubulin (11224, Proteintech, USA), β-actin (20536 Proteintech, USA) at 1:1000 dilution overnight and then with anti-rabbit HRP-linked IgG secondary antibody(7074, Cell signaling technology, USA) at 1:2000 dilution for 1 hour. The immunoreactive bands were visualized with ECL Ultra (New Cell and Molecular Biotech, Suzhou, China). α-tubulin and β-Actin was used as an internal control.

### *In vivo* lung metastasis assays

6-week-old BALB/c nude mice purchased from the Shanghai Laboratory Animal Center (Shanghai, China) were bred in SPF facilities. 1×10^6^ Cal27 cells transfected with luciferase-expressed lentivirus containing MFAP5 sequence or control lentivectors were injected into mice through lateral tail vein. Lung metastasis was monitored using bioluminescence imaging (BLI) by IVIS Imaging System once a week 23. The mice were killed at 5 weeks and the lung tissues were fixed and stained before microscopic analysis. The *in vivo* studies were approved by the Animal Care and Use Committee of Ninth People's Hospital, Shanghai Jiao Tong University School of Medicine.

### Statistical analysis

Statistical analysis was performed using SPSS 13.0 for Windows (SPSS Inc., Chicago, IL) and GraphPad Prism version 6 (GraphPad Software, San Diego, CA, USA) was used to plot the data. Student's t test and chi-square test was performed to assess the statistical significance of differences. Survival analysis was conducted using the Kaplan-Meier method and log-rank test. *P* < 0.05 was considered statistically significant (* *P* < 0.05, and ** *P* < 0.01). All values are expressed as the means ± standard error.

## Results

### Hypoxia induces MFAP5 expression in HNSCC cells

**Figure 1:**
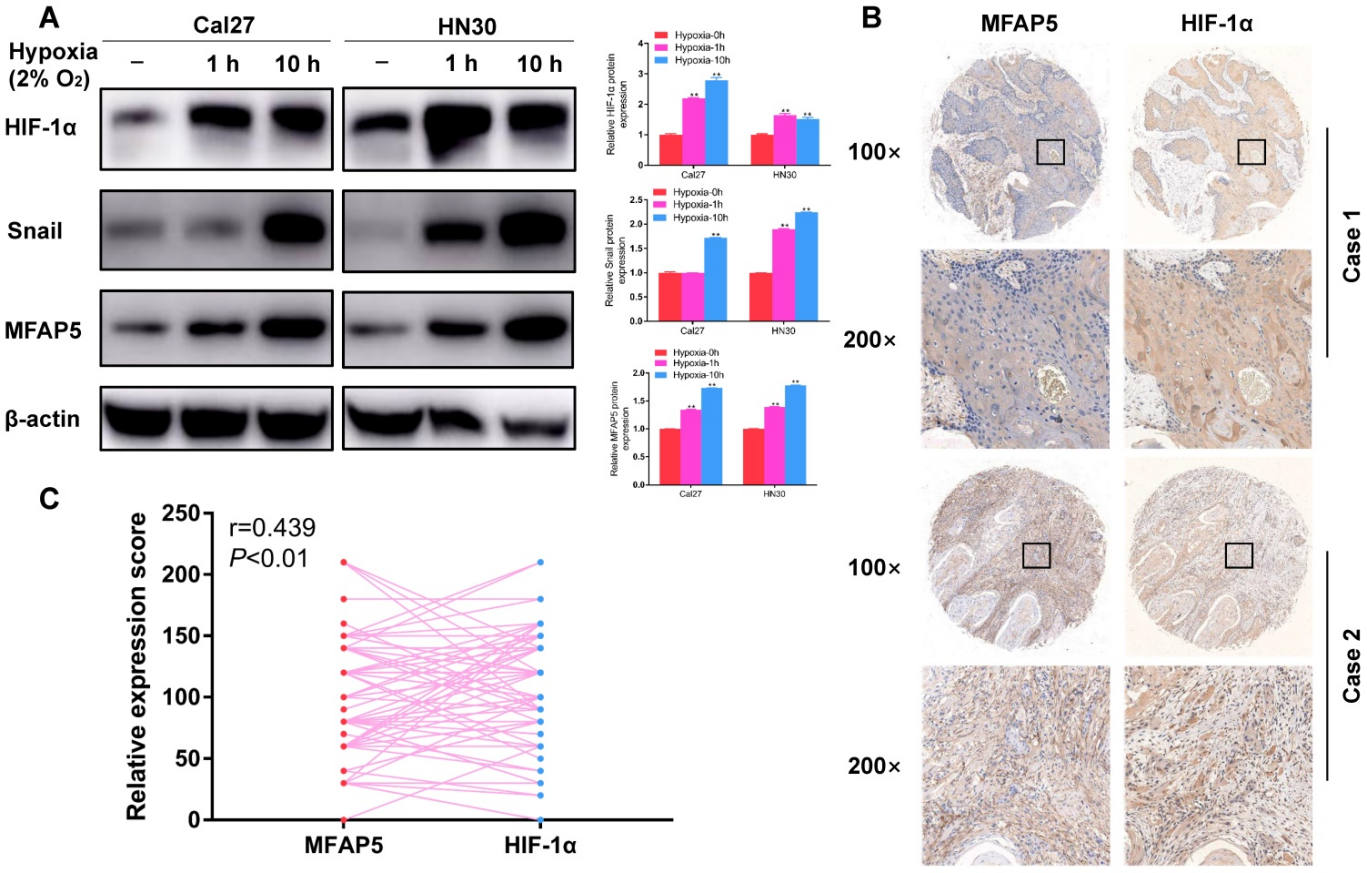
** Hypoxia induces MFAP5 expression in HNSCC.** (A) The protein level of HIF-1α, snail and MFAP5 in hypoxia environment for 0h, 1h and 10h. β-actin was used as an internal control. The proteins level is significant higher in hypoxia environment (**P<0.01). (B) The expression level of MFAP5 and HIF-1α is related in same tumor. (C) The correlation analysis of immunohistochemistry intensity for HIF-1α and MFAP5 in 84 patients' tissue sample (r=0.493, **P<0.01).

To testify the relationship between hypoxia and MFAP5 in HNSCC, we cultured the HNSCC cells in hypoxia environment for 1 h and 10 h, and compared the MFAP5 level by western blot. The result showed that MFAP5 expression was significant higher in hypoxia environment (Figure 1A, *P*<0.01), along with HIF-1α (Figure 1A, *P*<0.01), which indicated that hypoxia environment enhances the expression of MFAP5 in HNSCC. This conclusion has been verified in patients' tissue samples. The expression level of MFAP5 and HIF-1α was remarkably correlated in same tumor species (Figure 1B, 1C, *r*=0.493, *P*<0.01).

Interestingly, we found the expression of snail was also accord with MFAP5 (Figure 1A, *P*<0.01). As a key promotor of EMT progression, this result reminded us that MFAP5 might promote the expression of EMT phenotypic protein by activate snail, thus leading to tumor metastasis.

### MFAP5 promotes migration and invasion on HNSCC cell lines

**Figure 2:**
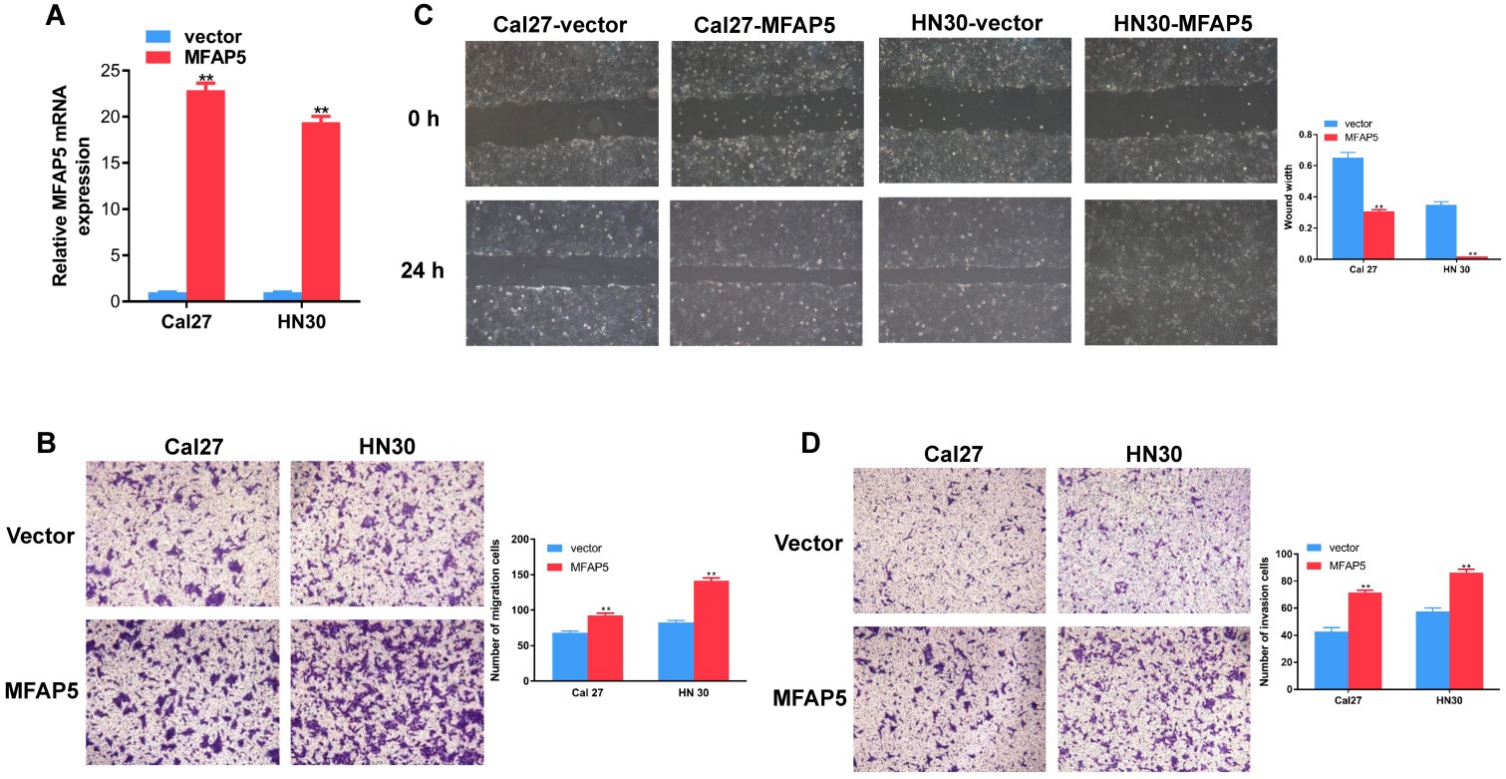
** Overexpressed MFAP5 promotes migration and invasion on HNSCC cell lines.** (A) Relative mRNA expression of MFAP5 in Cal27 and HN30 transfected with MFAP5 and vector (**P<0.01). (B) The migration assay was performed to testify the migration ability of Cal27 and HN30 transfected with MFAP5 or negative control lentivirus (**P<0.01). (C) The wound healing assay was performed to testify the migration ability of Cal27 and HN30 transfected with MFAP5 or negative control lentivirus (**P<0.01). (D) The invasion assay was performed to testify the invasion ability of Cal27 and HN30 transfected with MFAP5 or negative control lentivirus (**P<0.01).

To detect the function of MFAP5 in HNSCC, we transfected the MFAP5 lentivirus into Cal27 and HN30 cell lines and the result of PCR confirmed the transfection efficiency (*P*<0.01, Figure 2A). Transwell migration assay showed that overexpressed of MFAP5 markedly enhanced the motility of HNSCC cells (*P*<0.01, Figure 2B). The scratch wound healing assay confirmed this result. Cells transfected with MFAP5 lentivirus had a significant higher migration ability (*P* <0.01, Figure 2C). As a key parameter of metastasis, the cell invasion ability was assessed by transwell invasion assay. The result showed that cell invasion was enhanced by MFAP5 compared with control group (*P* <0.01, Figure 2D). The results above indicated that MFAP5 could promote migration and invasion in HNSCC cell lines.

### MFAP5 overexpression facilitates lung metastasis *in vivo*

**Figure 3:**
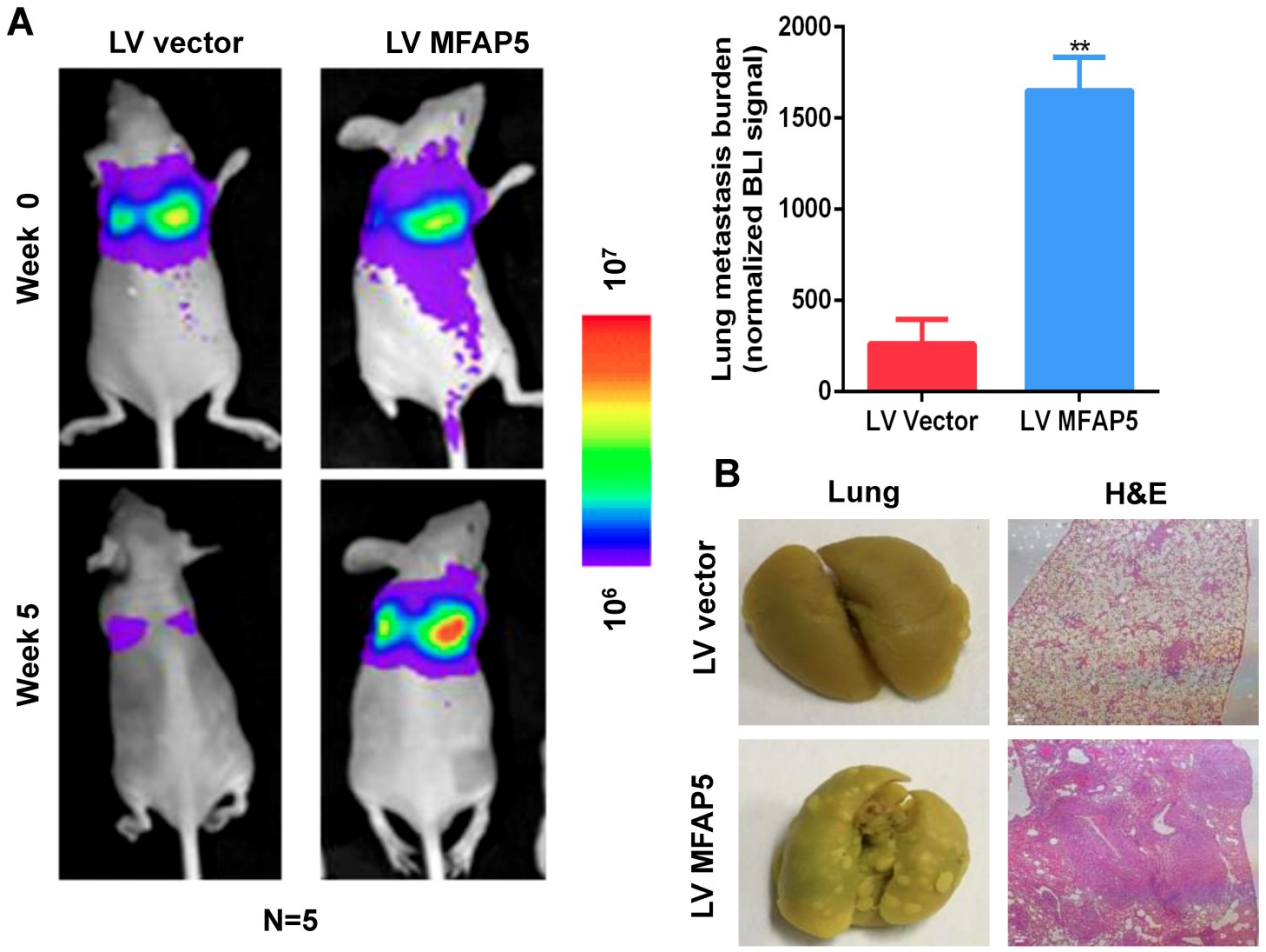
** Overexpressed MFAP5 facilitates lung metastasis *in vivo*.** (A) Bioluminescence imaging showed MFAP5 overexpression significantly increased lung metastasis burden (**P<0.01). (B) Gross specimen and H&E staining are shown (×100).

To examine the effect of MFAP5 on lung metastasis *in vivo*, we injected 1×10^6^ Cal27-MFAP5 into 6-week-old BALB/c nude mice with Cal27-vector as a control group. Bioluminescence imaging was used to examine lung metastasis, and H&E staining were performed to confirm lung metastasis nodules. Bioluminescence imaging results showed that MFAP5 overexpression can significantly increase lung metastasis burden (Figure 3A, *P*<0.01). This finding was verified by gross specimen and H&E staining (Figure 3B). Lung of Cal27-MFAP5 group had a higher amount of tumor node metastasis. The stained slice showed that the majority of lung tissue in MFAP5 group has been replaced by tumor cells. The results above proved that MFAP5 can facilitate metastasis in vivo.

### MFAP5 promotes the EMT program through AKT signaling pathway

**Figure 4:**
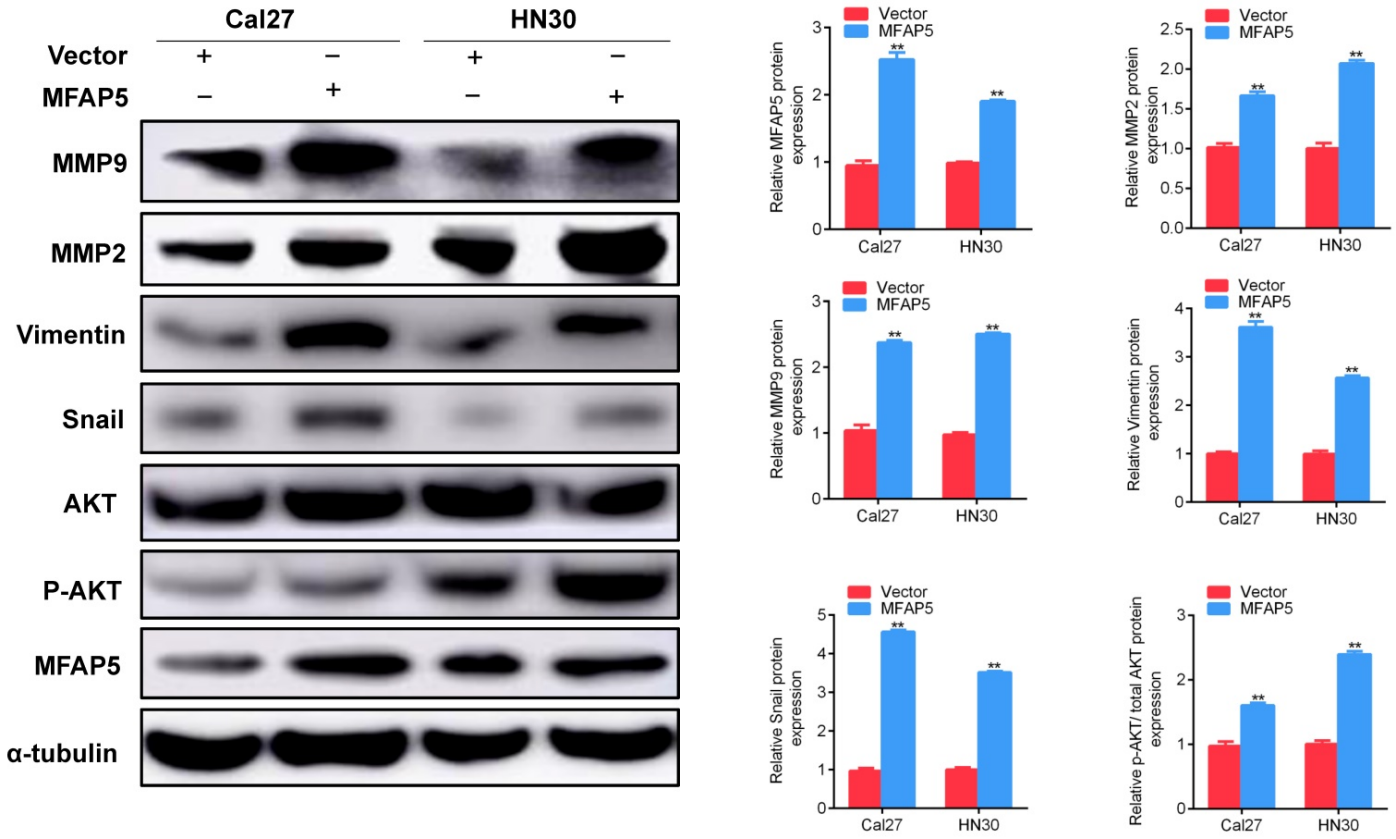
** Western blot was performed to detect the protein level of Cal27 and HN30 transfected with MFAP5 or negative control lentivirus.** The expression of MMP9, MMP2, vimentin, snail and p-AKT/AKT are significant higher along with MFAP5 (**P<0.01).

In accord with clinical observation, the results of *in vivo* and *in vitro* studies showed that MFAP5 promoted the migration and invasion of HNSCC. Combining with the fact that MFAP5 enhanced the expression of snail, we assumed that MFAP5 promotes tumor metastasis by activate the EMT program. To test this hypothesis, we firstly explored the protein level of MMP2, MMP9, vimentin and snail in HNSCC cells transfected with MFAP5 or vector. The results revealed that MFAP5 overexpression increased the level of these epithelium proteins (Figure 4). As a critical regulator of cell growth and migration, AKT signaling pathway was then detected to find out whether it was responsible for activating EMT program. The ratio of p-AKT/AKT in MFAP5 transfected cells was striking higher than control set *(P*<0.01, Figure 4). These results indicated that AKT phosphorylated by MFAP5 transported into nucleus, and activated snail to promote EMT program.

**Figure 5:**
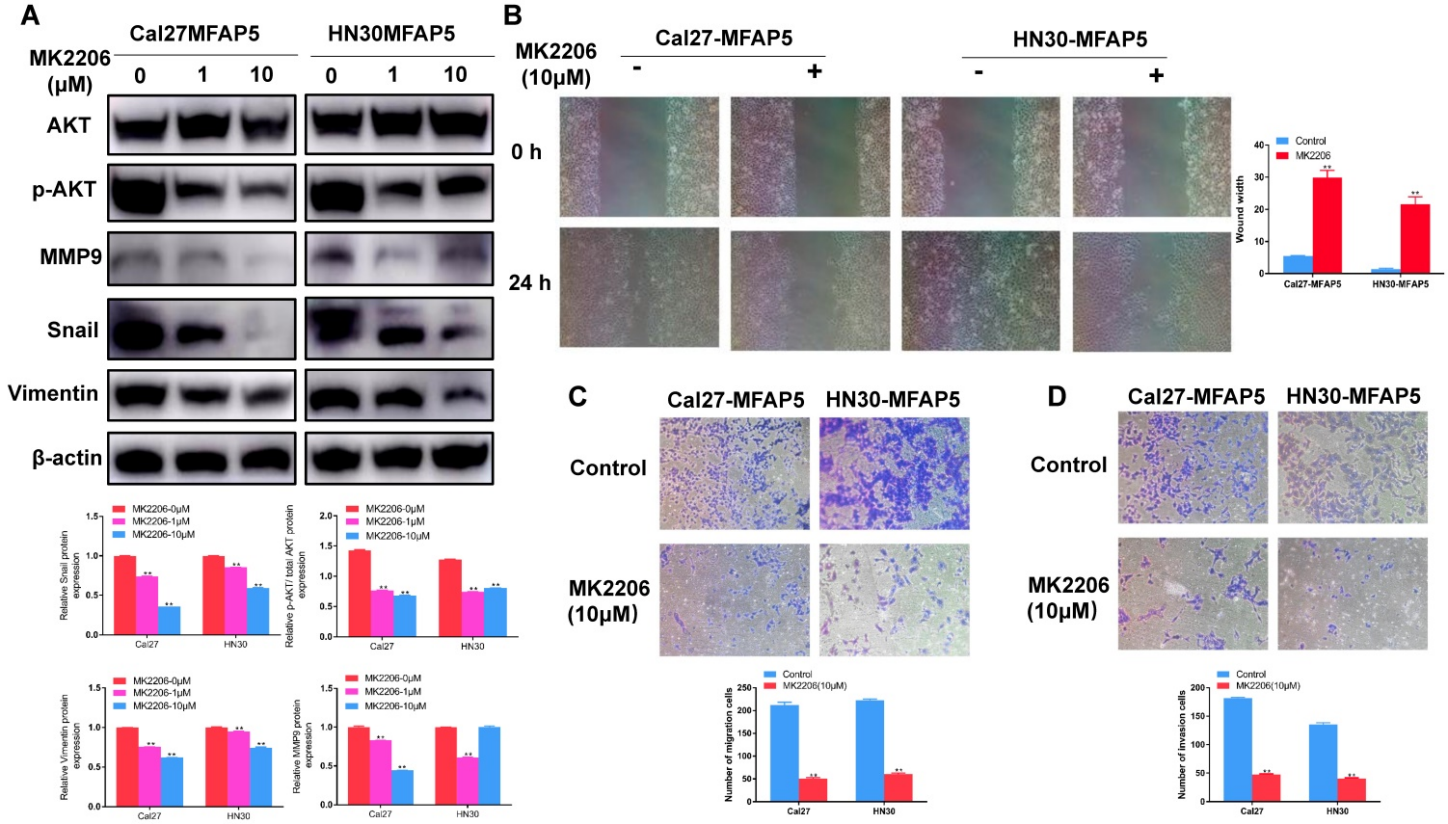
** The EMT program enhanced by MFAP5 could be reversed by MK2206.** (A) The protein level of MMP9, snail, vimentin and p-AKT/AKT was reduced after MK2206 was applied to Cal27-MFAP5 and HN30-MFAP5 (**P<0.01) (B) Wound healing assay was performed to Cal27-MFAP5 and HN30-MFAP5 with or without MK2206 (**P<0.01). (C) Migration assay was performed to Cal27-MFAP5 and HN30-MFAP5 with or without MK2206 (**P<0.01). (D) Invasion assay was performed to Cal27-MFAP5 and HN30-MFAP5 with or without MK2206 (**P<0.01).

To further prove the effect of AKT in this process, MK2206 (S1078, Selleck Chemicals, USA) was applied to inhibited the phosphorylation of AKT. Western blot showed that it could markedly reduce the expression of p-AKT (*P*<0.01, Figure 5A) in MFAP5 transfected cells, the level of MMP2, MMP9, vimentin and snail was declined along with it (*P*<0.01, Figure 5A). Cell invasion and migration ability which enhanced by MFAP5 were also inhibited by MK2206 (*P*<0.01, Figure 5B, 5C, 5D).

### Overexpression of MFAP5 correlates with poor prognosis and advanced stage in HNSCC

**Figure 6:**
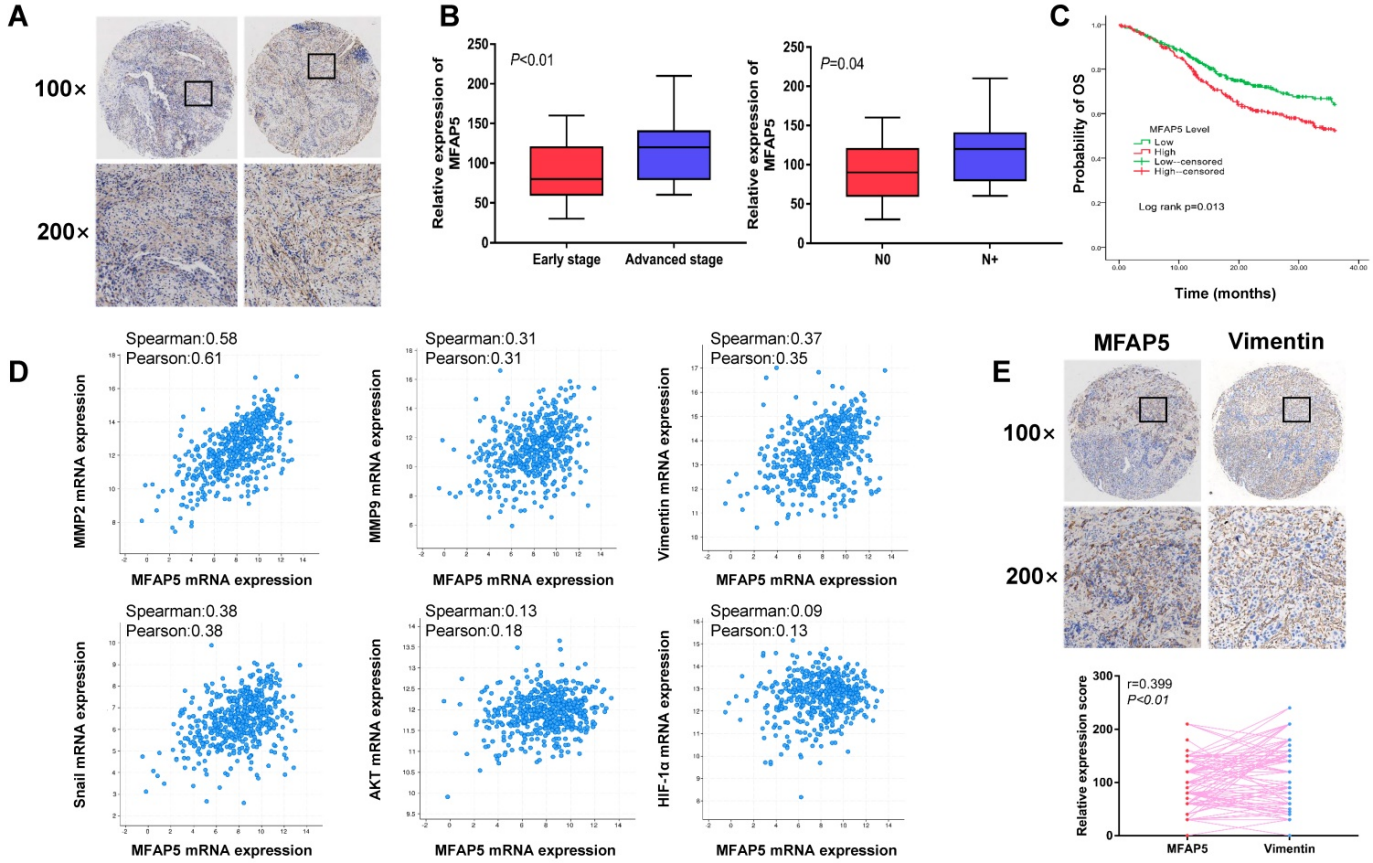
** Overexpression of MFAP5 can be observed in HNSCC and is associated with aggressive clinical features, poor outcome and EMT-related proteins.** (A) Overexpression of MFAP5 can be observed in HNSCC. (B) MFAP5 expression is related with advanced stage (*P*<0.01) and cervical lymph node metastasis (*P*=0.04). (C) TCGA data shows high level of MFAP5 lead to poor prognosis (*P*=0.013). (D) Correlation analysis of relative mRNA expression shows MFAP5 is associated with EMT-related proteins. (E)The correlation analysis of immunohistochemistry intensity for vimentin and MFAP5 in 84 patients' tissue sample (r=0.399, *P*<0.01).

Eighty-four tissue samples were performed immunohistochemical staining to determine the expression of MFAP5 in HNSCC. In consistent with our previous study, MFAP5 has a high expression inside HNSCC tumor tissue (Figure 6A). Combining with the clinical data of patients, we also discovered that MFAP5 expression was related with advanced stage and cervical lymph node metastasis of HNSCC (*P*<0.01, Figure 6B), which are important prognostic indicators. However, age, sex, site and pathologic grade were not related with MFAP5 expression level (Table 1). These results were further validated by The Cancer Genome Atlas (TCGA) dataset. Patients with overexpression of MFAP5 have a significant worse prognosis compared with low-expression set (*P*=0.013, Figure 6C). Besides, correlation analysis from TCGA dataset revealed that MFAP5 expression was correlated with MMP2, MMP9, vimentin, snail, AKT and HIF-1α which were consistent with our findings (Figure 6D). In situ staining of tissue samples indicated that high expression of MFAP5 could led to a high level of vimentin (*P*<0.01, Figure 6E). The results from tissue samples and dataset further demonstrated that MFAP5 promoted EMT program via AKT pathway in HNSCC.

## Discussion

HNSCC as a common cancer can elicit both physical and mental damage in patients. Given that the long-term survival of HNSCC remains poor, an effective biomarker is imperative, especially for metastasis 24, 25. As many studies suggest, hypoxia is a universal phenomenon in tumor microenvironment and it makes a major contribution to metastasis. EMT program is also believed to have an important role in initiate metastasis 26, but the molecular mechanism of EMT in HNSCC remains unclear. Our previous study has already demonstrated that overexpression of MFAP5 significantly correlated with cervical lymphatic metastasis and prognosis in HNSCC patients 15. In the current study, we demonstrated that MFAP5 plays a critical role in hypoxia induced EMT program.

The relationship between MFAP5 and poor prognosis has been proven in many cancers as well as HNSCC 27-29. In addition, we demonstrated that MFAP5 was also correlated with advanced stage and metastasis in this study. As a common phenomenon in tumor, EMT process is believed to have great impact on metastasis. EMT process provides tumor cell the ability to elude adhesion by decompose tumor stroma and change cellular morphology. Those evidences give us a hint that MFAP5 may promote metastasis by enhance the expression on phenotypic protein of EMT. Our results proved this hypothesis. MMP2, MMP9 and vimentin have a markedly higher expression in MFAP5 transfected cells, and tissue samples verified this finding.

Hypoxia is another important factor for metastasis yet the correlation of MFAP5 and hypoxia is rarely reported. As the indicator and product of hypoxia environment, the expression of HIF-1α increase the extravasation of cancer cell and recruitment of circulating tumor cell. Nonetheless, the underlying mechanism remains unclear. Most previous studies claimed that MFAP5 is majorly secreted by stroma cells like cancer-associated fibroblasts (CAFs). Our research demonstrated for the first time that this lack oxygen environment could stimulate tumor cell to secret MFAP5. This finding suggests that MFAP5 is an important intermediate in hypoxia leading to metastasis.

It has been proven that MFAP5 exerts its function by binding the RGD domain on cancer cell to αVβ3 integrin whereas the downstream is still diverse 30. Leung et al. discovered that binding αVβ3 integrin could activate FAK/CREB signaling pathway and increase the expression of TNNC1 which would lead to poor prognosis 31; Wu et al. claimed that MFAP5 promote EMT program in breast cancer by activated TGF-β/Notch pathway 32. Our research proved that AKT pathway is a key downstream for MFAP5 in HNSCC. The activation of AKT has a huge impact on tumor growth, proliferation, migration and invasion 33. Yamamoto et al. found that AKT pathway was closely related with lung cancer proliferation and the migration ability would significant inhibited by down regulate AKT in NSCLC cells 34. Previous studies have nicely demonstrated its function for promoting tumor progression. However, in this study, further exploration showed that AKT enhances the malignant biological behavior of tumor by increases the expression of snail, which is a promotor of EMT progress. The over-expressed snail facilitates the expression of MMP2, MMP9 and vimentin, which leads to the enhancement of invasion and migration. Data from an independent cohort further proved this finding. This result not only explored the mechanism of MFAP5 in HNSCC, but also provided a new target for the future treatment and prognosis prediction.

However, current studies of MFAP5 are majorly focus on its impact for tumor cells and the critical role of tumor microenvironment is often overlooked. Tumor microenvironment can not only secrete proteins that directly affect tumor cells like MFAP5, but also influence the prognosis by constructing tumor matrix and tumor immunity. For this reason, fully understanding the functions of these proteins on tumor-associated-stromal cells, such as infiltrating lymphocytes, CAFs, may make a big contribution to future treatment and prognosis. Therefore, the impact of MFAP5 on tumor environment cells is a promising future direction.

For the first time, we linked hypoxia, MFAP5 and metastasis into one process and proved that MFAP5 played an important role in hypoxia induced tumor progress in HNSCC. However, there are still some unsolved problems that require future exploration. First, whether the AKT pathway is directly activated by αVβ3 integrin is still unclear. Second, after the stimulation of hypoxia and overexpression of MFAP5 in the first place, what is the feedback mechanism of cancer cell? Will the overexpressed MFAP5 inhibit or promote its further secretion? Lastly, the interaction between MFAP5 and other cytokines is still unclear. Therefore, further studies are still needed to explore the underlying mechanism and novel downstream of MFAP5. Nonetheless, the critical role of MFAP5 in HSNCC progression for its enhancement of cell migration and invasion is undoubted. The findings of this study nicely proved MFAP5 as a potential biomarker and therapeutic target for HNSCC. Drugs targeting MFAP5 may be an effective treatment in future clinic application.

## Conclusion

In conclusion, our study observes that MFAP5 has a significant higher expression in HNSCC patients and is correlated with advanced stage and cervical lymphatic metastasis. We further demonstrate that hypoxia can induce the expression of MFAP5 in HNSCC cell, which then promotes cell migration and invasion through AKT pathway. Overall, HIF1α-MFAP5-EMT axis will be a very promising therapeutic target to abolish metastasis in HNSCC.

## Figures and Tables

**Table 1:** Baseline data of 84 patients enrolled

Variable	No. (%)	Low level		High level	*P*
		No. (%)		No. (%)	
**Age**					0.382
<60	40 (47.6%)	18 (41.9%)		22 (53.7%)	
≥60	44 (52.4%)	25 (58.1%)		19 (46.3%)	
**Sex**					0.279
Male	48 (57.1%)	22 (51.2%)		26 (63.4%)	
Female	36 (42.9%)	21 (48.8%)		15 (36.6%)	
**Site**					0.886
Tongue	37 (44.0%)	18 (41.9%)		19 (46.3%)	
Gingival	13 (15.5%)	7 (16.3%)		6 (14.6%)	
Buccal mucosa	15 (17.9%)	7 (16.3%)		8 (19.5%)	
Floor of the mouth	10 (11.9%)	6 (14.0%)		4 (9.8%)	
Hard palate	5 (6.0%)	2 (4.7%)		3 (7.3%)	
Lip	4 (4.8%)	3 (7.0%)		1 (2.4%)	
**TNM stage**					
Early stage	37 (44.0%)	28 (65.1%)		9 (22.0%)	***<0.01***
Advanced stage	47 (56.0%)	15 (34.9%)		32 (78.0%)	
**pN stage**					
N0	51 (60.7%)	31 (72.1%)		20 (48.8%)	***0.044***
N+	33 (39.3%)	12 (27.9%)		21 (51.2%)	
**Pathology grade**					0.377
Well-differentiated	36 (42.9%)	21 (48.8%)		15 (36.6%)	
Moderate-differentiated	32 (38.1%)	16 (37.2%)		16 (39.0%)	
Poorly-differentiated	16 (19.0%)	6 (14.0%)		10 (24.4%)	

## Abbreviations

HNSCC: Head and neck squamous cell carcinoma
MFAP5: Microfibrillar-associated protein 5
MAGP2: Microfibril-associated glycoprotein 2
EMT: epithelial-mesenchymal transition
HIF-1α: Hypoxia-inducible factor-1α
DMEM: Dulbecco's modified Eagle's medium
PBS: phosphate buffered saline solution
SDS-PAGE: sodium dodecyl sulfate-polyacrylamide gel electrophoresis
PVDF: polyvinylidene fluoride
TCGA: The Cancer Genome Atlas
CAFs: cancer-associated fibroblasts
